# In Vivo Imaging of Flavoprotein Fluorescence During Hypoxia Reveals the Importance of Direct Arterial Oxygen Supply to Cerebral Cortex Tissue

**DOI:** 10.1007/978-1-4939-3023-4_29

**Published:** 2015-06-22

**Authors:** K. I. Chisholm, K. K. Ida, A. L. Davies, D. B. Papkovsky, M. Singer, A. Dyson, I. Tachtsidis, M. R. Duchen, K. J. Smith

**Affiliations:** 1 University College London, London, UK; 2 University of São Paulo, São Paulo, Brazil; 3 University College Cork, Cork, Ireland

**Keywords:** Oxygen, Mitochondria, Brain, Vasculature, Confocal microscope

## Abstract

Live imaging of mitochondrial function is crucial to understand the important role played by these organelles in a wide range of diseases. The mitochondrial redox potential is a particularly informative measure of mitochondrial function, and can be monitored using the endogenous green fluorescence of oxidized mitochondrial flavoproteins. Here, we have observed flavoprotein fluorescence in the exposed murine cerebral cortex in vivo using confocal imaging; the mitochondrial origin of the signal was confirmed using agents known to manipulate mitochondrial redox potential. The effects of cerebral oxygenation on flavoprotein fluorescence were determined by manipulating the inspired oxygen concentration. We report that flavoprotein fluorescence is sensitive to reductions in cortical oxygenation, such that reductions in inspired oxygen resulted in loss of flavoprotein fluorescence with the exception of a preserved ‘halo’ of signal in periarterial regions. The findings are consistent with reports that arteries play an important role in supplying oxygen directly to tissue in the cerebral cortex, maintaining mitochondrial function.

## Introduction

Mitochondrial pathology has been implicated in a wide range of diseases, including multiple sclerosis [1], Parkinson’s disease [2], and sepsis [3], emphasizing the need for greater understanding of the role of mitochondrial function in vivo.

The electron transport chain (ETC) is one of the main regulators of mitochondrial function, and can be indirectly assessed using confocal microscopy and membrane potential-sensitive dyes, such as tetramethylrhodamine methyl ester (TMRM), or endogenous fluorescent indicators of ETC redox potential, including oxidized flavoproteins and reduced nicotinamide adenine dinucleotide (NAD(P)H). Reduced NAD(P)H and oxidized flavoproteins have fluorescent properties [4] that differ from those of their oxidized and reduced counterparts, respectively, permitting cellular redox potential to be mapped with the spatial and temporal resolution afforded by confocal microscopy. ETC efficiency depends on oxygen availability; therefore hypoxic conditions can lead to reduction of the ETC due to accumulation of electrons and this can be visualized using flavoprotein and/or NAD(P)H fluorescence [5].

Oxygen is normally supplied at a rate sufficient to maintain tissue levels above the critical value necessary for mitochondrial function [6], and this supply has historically been attributed to the capillary network [7]. However, more recent evidence suggests that substantial oxygen diffusion can also occur across arteries and arterioles [8–11].

Here we examined mitochondrial function in vivo, as assessed by endogenous flavoprotein fluorescence, in response to changes in the inspired oxygen fraction (FiO_2_) to explore the role of arteries in the supply of cortical tissue oxygen.

## Method

C57bl/6 mice (~20 g) were housed in a 12 h light/dark cycle with food and water *ad libitum*. All experiments were performed in accordance with the UK Home Office Animals (Scientific Procedures) Act (1986).

Mice were anaesthetised (~2 % isoflurane in room air, or 2 g/kg urethane and 20 mg/kg ketamine i.p.; the injectable anaesthetic was only used when imaging NAD(P)H together with flavoproteins), and placed on a homeothermic heating mat to maintain rectal temperature at 37 °C. An incision was made in the scalp, the skull cleaned of connective tissue, and affixed to a titanium bar for stability with dental cement (Contemporary Ortho-Jet Powder, USA) mixed with cyanoacrylate glue (Loctite, Henkel Ltd., UK). The cortex was exposed by partial craniotomy (~5 mm diameter) over the right hemisphere, and the dura was moistened and cleaned with saline. A circular glass coverslip (6 mm) was placed over a ring of petroleum jelly to prevent evaporation during imaging. In a subset of experiments, oxygen-sensitive microbeads impregnated with a phosphorescent dye, PtPFPP (ex: 543 nm; em: 650 nm, collected with 585 nm long pass filter) were spread on the dura (5 μl of 5 mg/ml aqueous suspension) prior to placement of the coverslip. Alternatively, TMRM (T-668, Molecular Probes, Invitrogen, UK; 1 μM incubated on the cortex for 30 min; ex 561 nm, em 584–656) was applied after removal of the dura. Following surgery, the animals were moved to a custom-made stage for confocal microscopy.

In experiments employing cyanide (NaCN) or carbonyl cyanide 4-(trifluoromethoxy)phenylhydrazone (FCCP), a coverslip was not used, and the dura was removed. A well was created around the exposed cortex using silicone (Body Double, Smooth-On Inc., USA) and filled with 40 μl saline to which 2 μl of NaCN or FCCP (working concentration of 5 mM and 10 μM, respectively) were added during time lapse imaging. Five of these images were averaged, seconds or minutes after application, depending on the stabilization of the image. FiO_2_ was controlled by mixing oxygen and nitrogen as indicated (100, 21, 15, 21, 10, 21 and 5 % oxygen, each for 5 min).

The endogenous flavoprotein signal (ex: 488 nm, em: 505–570 nm) was imaged with a LSM 5 Pascal laser-scanning confocal microscope (Zeiss, Germany), using time series recordings with an in-plane resolution of 512 by 512 pixels and an optical slice thickness of 896 μm. Endogenous NAD(P)H (ex: 720, em: 430–480) was imaged using a Zeiss 510 NLO META equipped with a Coherent Chameleon Ti:sapphire laser.

Images were processed using Fiji/ImageJ Version 1.48v. Time lapse sequences were aligned using the ‘Stackreg’-Plugin. Statistical significance was assessed using the IBM SPSS Statistics 22 package.

## Results

Under normoxic conditions, endogenous green fluorescence was uniformly distributed across the surface of the cerebral cortex, with the superficial vasculature clearly defined in negative contrast. Arteries were distinguishable from veins based on their morphology, and their uniform outline, which was typically highlighted by brightly fluorescent walls.

**Fig. 29.1:**
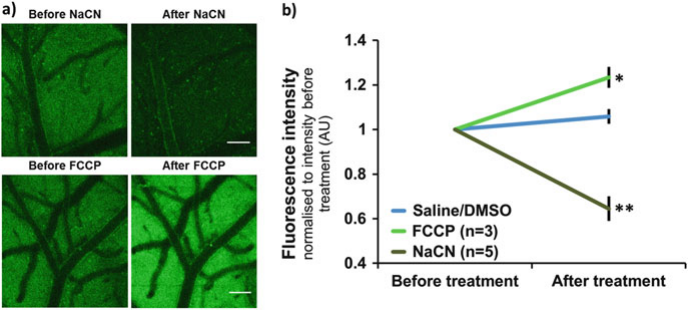
(**a**) Fluorescence intensity in response to NaCN and FCCP. Scale bar = 100 μm. (**b**) Quantification of fluorescence intensity before and after application of saline/DMSO or NaCN/FCCP to the cortex. Data are normalised to signal intensity before treatment and displayed as mean ± SEM. Statistical significance was assessed using a paired sample t-test (*p ≤ 0.05, **p ≤ 0.01)

The origin of endogenous green fluorescence was explored by administrating agents known to change the redox state of flavoproteins. Application of NaCN (reducing the ETC) significantly decreased fluorescence intensity (~35 %), whereas application of FCCP (oxidizing the ETC) significantly increased fluorescence intensity (~23 %; Fig. 29.1). These data are consistent with the assumption that green autofluorescence originates from oxidized mitochondrial flavoproteins.

**Fig. 29.2:**
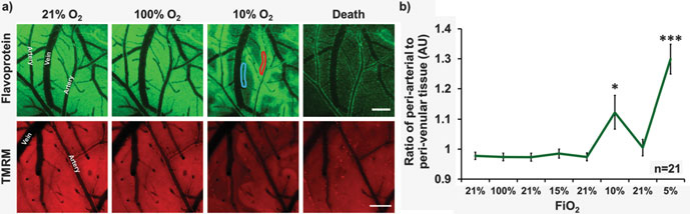
(**a**) Flavoprotein (*green*) and TMRM (*red*) fluorescence in response to changes in FiO_2_. Scale bar = 200 μm. (**b**) The ratio of periarterial to perivenular tissue flavoprotein fluorescence intensity (examples indicated in **a**), *red * = periarterial and *blue* = perivenular. Data are displayed as mean ± SEM. Statistical significance was assessed using a paired sample t-test (*p ≤ 0.05, ***p ≤ 0.001)

Although increasing FiO_2_ had no effect on the signal, reducing FiO_2_ (to ≤ 10 %) resulted in a marked decrease in flavoprotein fluorescence. This decrease preferentially affected tissue distal to arteries, with a ‘halo’ of preserved fluorescence in tissue adjacent to arteries and arterioles (Fig. 29.2), and typically appeared at an FiO_2_ of 5–10 %.

To explore whether changes in flavoprotein fluorescence were associated with changes in mitochondrial membrane potential, we examined the effects of hypoxia on TMRM fluorescence. The same arterial ‘halos’ were observed with TMRM at 5–10 % FiO_2_ as were seen when imaging flavoproteins (Fig. 29.2a).

**Fig. 29.3:**
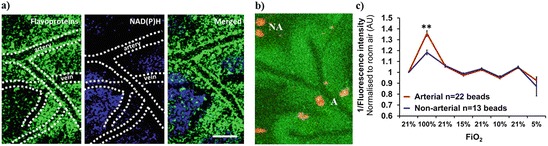
(**a**) Flavoprotein (*green*) and NAD(P)H (*blue*) fluorescence in response to hypoxia. (**b**) Oxygen-sensitive phosphorescent beads (*red*) on the hypoxic cortex. A = Periarterial, NA = Nonarterial. (**c**) Bead emission intensity in response to changes in FiO_2_. Data are displayed as mean ± SEM. Statistical significance was assessed using an independent sample t-test (**p≤0.01)

The changes in the distribution of the flavoprotein fluorescence at reduced FiO_2_ varied inversely with NAD(P)H fluorescence (Fig. 29.3a). As expected, changing the FiO_2_ also resulted in corresponding changes in cortical tissue oxygen concentration, as measured by oxygen-sensitive phosphorescent beads (Fig. 29.3b). When FiO_2_ was increased, a larger change in emission intensity was observed in beads within the ‘halo’ of preserved flavoprotein fluorescence surrounding arteries than beads located distal to arteries (Fig. 29.3c). At an FiO_2_ of 5 %, a greater response in fluorescence was observed in beads in nonarterial regions, but this difference was not significant (Fig. 29.3c).

## Discussion

Using endogenous flavoprotein fluorescence we have demonstrated that cortical mitochondrial function is selectively impaired at low FiO_2_, with preservation of mitochondrial redox potential around arteries and arterioles, demonstrating the role of these structures in the direct supply of oxygen to cerebral cortex tissue.

The use of flavoprotein fluorescence as an indicator of mitochondrial redox potential is well established [4, 12–14] but its application in vivo has been limited. To our knowledge flavoprotein autofluorescence has not been used previously to assess cortical redox state in response to changes in FiO_2_ at the high spatial and temporal resolutions of confocal microscopy.

To confirm the mitochondrial origin of the flavoprotein signal, we assessed signal changes in response to variations in mitochondrial redox state, induced by the well characterised agents NaCN and FCCP. The signal source was further validated by simultaneous measurement of flavoprotein and NAD(P)H fluorescence, revealing an inverse relationship and permitting mapping of the redox ratio of the cerebral cortex in vivo.

Under normoxic conditions, oxygen supply to the brain was sufficient to maintain functioning mitochondria throughout the cortex. Increasing FiO_2_ accordingly had no effect on flavoprotein fluorescence, presumably because oxygen availability was not a rate limiting factor in the function of the ETC.

A slight decrease in FiO_2_ to 15 % also had little influence on the mitochondrial redox potential. However, a further decrease of FiO_2_ to ≤ 10 % induced a characteristic change, with preservation of oxidized flavoprotein in periarterial tissue, and reduction of flavoproteins in distal tissue and near veins. This pattern was also seen with TMRM, suggesting other measures of mitochondrial function such as membrane potential are also affected. Preservation of flavoprotein and TMRM fluorescence around arteries is not consistent with the historical assumption that oxygen exchange is limited to capillaries [7]. Rather, our data support recent evidence that arteries play a major role in supplying cortical oxygen directly to tissue [8–11].

As expected, decreasing FiO_2_ also decreased cortical oxygenation as measured by oxygen-sensitive beads. Tissue oxygenation in periarterial regions increased to a greater extent during hyperoxia than in nonarterial areas, further supporting the suggestion that oxygen exchange occurs along arteries [8–11]. However, no measurable difference in periarterial and nonarterial tissue responsiveness was detected at ≤10 % FiO_2_, despite the decrease in oxidized flavoprotein remote from arteries.

In conclusion, changes in mitochondrial redox potential, as demonstrated by a regionally selective decrease in flavoprotein fluorescence, are evident in the hypoxic cerebral cortex. The simultaneous preservation of oxidized flavoproteins in tissue surrounding arteries is consistent with the direct delivery of oxygen from arteries to adjacent tissue.
